# A cost-effectiveness analysis of risk-based intervention for prevention of cardiovascular diseases in IraPEN program: A modeling study

**DOI:** 10.3389/fpubh.2023.1075277

**Published:** 2023-02-24

**Authors:** Amirparviz Jamshidi, Rajabali Daroudi, Eline Aas, Davood Khalili

**Affiliations:** ^1^Prevention of Metabolic Disorders Research Center, Research Institute for Endocrine Sciences, Shahid Beheshti University of Medical Sciences, Tehran, Iran; ^2^Department of Health Management and Health Economics, University of Oslo, Oslo, Norway; ^3^Department of Health Management, Policy and Economics, School of Public Health, Tehran University of Medical Sciences, Tehran, Iran; ^4^Department of Biostatistics and Epidemiology, Research Institute for Endocrine Sciences, Shahid Beheshti University of Medical Sciences, Tehran, Iran

**Keywords:** cost effectiveness, cardiovascular risk factors, cardiovascular diseases, modeling, diabetes preventing program, CVD prevention, primary health care, WHO PEN

## Abstract

**Background:**

IraPEN, a program developed in Iran based on the World Health Organization (WHO) package of essential noncommunicable (PEN) disease interventions for primary healthcare, was launched in 2015. Preventive interventions for cardiovascular diseases (CVDs) are based on the level of risk calculated using the WHO CVD risk chart.

**Objective:**

The main objective of this study was to measure the potential cost-effectiveness (CE) of IraPEN preventive actions for CVD in comparison with the *status quo*.

**Methods:**

A CE analysis from a healthcare perspective was conducted. Markov models were employed for individuals with and without diabetes separately. Based on the WHO CVD risk chart, four index cohorts were constructed as low (<10%), moderate (10%−19%), high (20%−29%), and very high risk (≥30%). Life years (LY) gained and quality-adjusted life years (QALY) were used as the outcome measures.

**Results:**

The intervention yields an incremental cost-effectiveness ratio (ICER) of $804, $551, and –$44 per QALY for moderate, high, and very high CVD risk in groups without diabetes, respectively. These groups gained 0.69, 0.96, and 1.45 LY, respectively, from the intervention. The results demonstrated an ICER of $711, $630, –$42, and –$71 for low, moderate, high, and very high-risk groups with diabetes, respectively, while they gained 0.46, 1.2, 2.04, and 2.29 years from the intervention.

**Conclusion:**

The IraPEN program was highly cost-effective for all CVD risk groups in the individuals without diabetes except the low-risk group. The intervention was cost-effective for all patients with diabetes regardless of their CVD risk. The results demonstrated that the IraPEN program can likely provide substantial health benefits to Iranian individuals and cost savings to the national healthcare provider.

## Background

Cardiovascular diseases (CVDs) are the leading cause of death worldwide and people die from CVDs more than any other causes. CVDs are considered a development issue as almost 75% of global CVD deaths occur in low- and middle-income countries. However, the majority of CVDs can be prevented by reducing the burden of risk factors (1).

Iran with an 80 million population in 2016, as an upper-middle-income developing country, has acquired many achievements in the public health sector during the past three decades. A well-balanced referral system within a broad PHC network, even in far stretches of villages, could provide access to healthcare for 95% of the community and control communicable diseases efficiently. This accomplishment resulted in a life expectancy of more than 75 years for men and more than 77 years for women. At present, the transition to chronic and noncommunicable diseases (NCDs) including CVDs, cancer, and mental disorders is the main problem in the health system. Based on the last report by the World Health Organization (WHO), NCDs are estimated to account for almost 80% of total deaths in Iran, while almost half of them (43%) are caused by CVDs. The comparison of Iranians' CVD mortality rate with other countries shows that not only it is substantially higher than high-income countries but also it is much higher than the countries in the region (2).

During the last two decades, many positive actions (e.g., public education, opportunistic finding of diabetic and hypertensive cases in network system, etc.) have been carried out to control NCDs in the country. Despite major achievements, NCDs and their subsequent burden have increased in the country (3).

In 2010, the WHO launched the package of essential noncommunicable (PEN) disease intervention for primary care in low-resource settings to deliver an adequate quality of care and, consequently, reduce the burden of these diseases in developing countries. WHO PEN has effective tools to facilitate early diagnosis and management of CVD, chronic respiratory diseases, diabetes, and cancer to prevent their upcoming morbidities and premature mortalities (e.g., stroke, myocardial infarction, renal failure, blindness, amputations, etc.) (4).

In 2015, IraPEN, an adaptation of WHO PEN, was launched as a part of the national Healthcare Reform Plan in Iran. Providing universal healthcare coverage and access to NCD prevention and treatment for all were the main goals of this reform. The first phase of the IraPEN program had been piloted in four cities, and the results were promising. In 2018, the program was expanded to all provinces of Iran. It was expected that at this phase, the program would cover up to four million people, and then based on the results, it would be expanded nationwide (4). Due to the IraPEN project size and its impact on the national healthcare budget, it is essential to make a detailed evaluation of these pilot enforcements to pave the way for IraPEN national implementation. Therefore, there is a need for an economic analysis that can estimate costs and effects as well as an incremental cost-effectiveness ratio (ICER) of the IraPEN program in comparison with the *status quo* (no prevention). Therefore, the main objective of this analysis is to measure the cost per life-year (LY) gained as well as the cost per quality-adjusted life years (QALY) gained by the IraPEN program.

## Methods

This study evaluates the potential cost-effectiveness (CE) of the IraPEN program in comparison to the *status quo* through a health economic evaluation and the outcomes expressed in terms of QALY and LY gained for each CVD risk group. The target group of this analysis is all Iranian people aged older than 40 years and the evaluated intervention is the same as the recommended intervention of WHO PEN which is included screening, monitoring, and medications.

### Model structure

**Figure 1:**
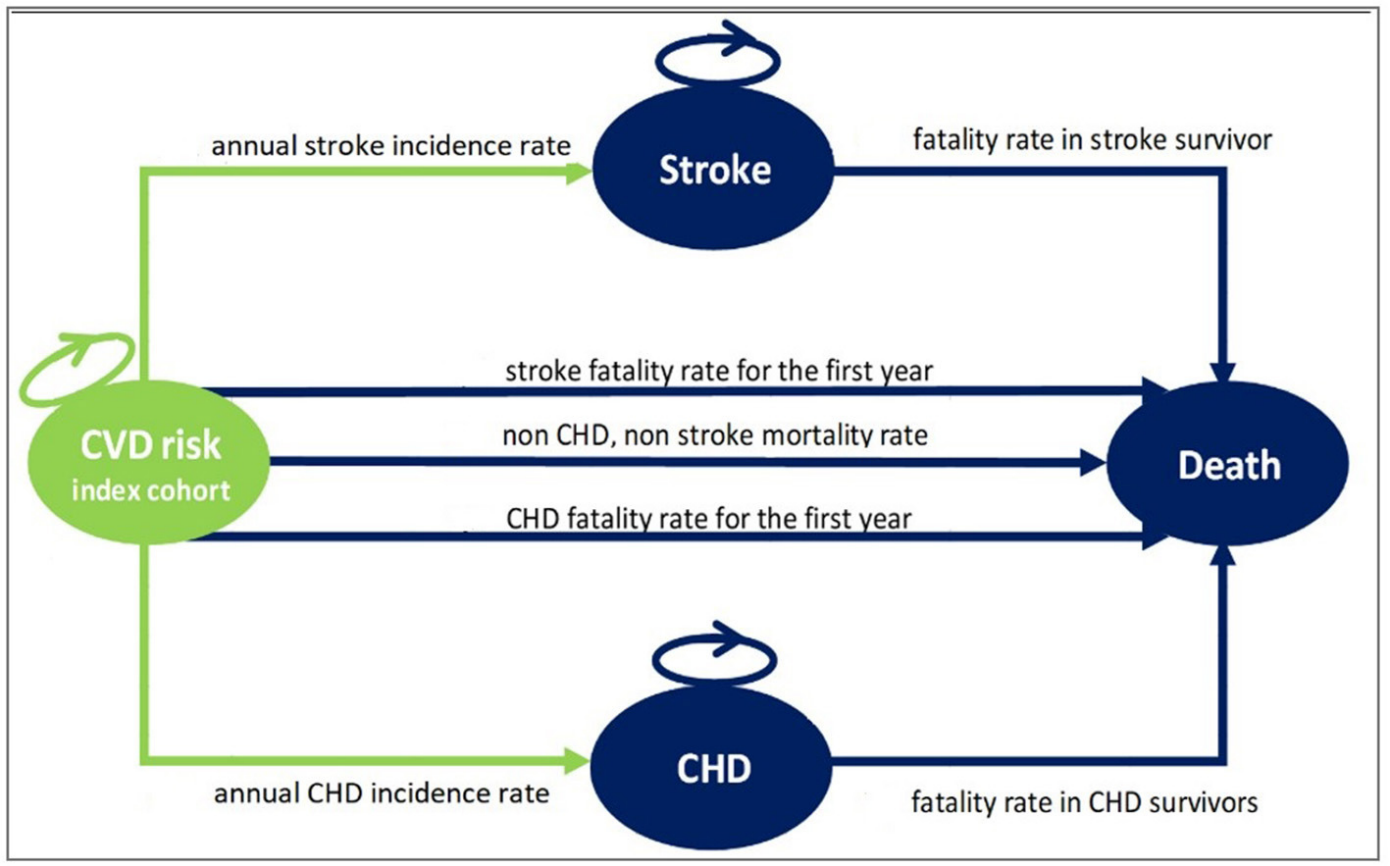
The structure of the Markov model used for the IraPEN analysis. CVD risk index cohort state, healthy individuals with different CVD risk; Stroke state, alive individuals after the first stroke event; CHD state, alive individuals after the first CHD event; Death state, dead individuals.

Two separate Markov decision models were developed to compare the long-term costs and health benefits of the IraPEN program (primary CVD prevention) with the *status quo* (no prevention) in two distinct scenarios. In the base case scenario, individuals without diabetes were included, while patients with diabetes were included in the alternative scenario. Each Markov model has four health states with transitions between the states according to age, sex, and the CVD risk characteristics of participants (Figure 1). In contrast to the usual Markov models, which are structured based on cohorts with average profiles, we decided to categorize the individuals based on their CVD risks. As the intervention (treatment) varied according to CVD risk level, it is logical to model them separately. In this way, we can take into account their specific characteristics. Therefore, based on WHO/ISH CVD risk prediction charts for EMR B, four index cohorts were constructed (5). These hypothetical cohorts were used as a representative for individuals with low, moderate, high, and very high CVD risk profiles. The CVD risk state represents the starting point for all people who are 40 years old. It was assumed that people in this state may either remain in the same health state, move to the stroke state, or CHD (coronary heart disease) state, or die. As long as they are event-free, these individuals can stay in a healthy state, but after the first event, they move to the CHD or stroke state and stay there until their death.

In WHO/ISH CVD risk prediction charts, the CVD risk is calculated based on individuals' age and risk factors such as blood pressure, lipid profile, diabetes, and smoking status and categorized into the following five groups: below 10% (low-risk group), between 10 and 19% (moderate-risk group), between 20 and 29% (high-risk group), between 30 and 39%, and above 40% (very high-risk group). As the individuals in the two latter groups are treated the same, in the IraPEN program, whoever has a CVD risk above 30% is categorized as the very high-risk group.

Therefore, considering what was mentioned earlier, all the Iranians aged older than 40 years who did not have CHD or stroke events before were eligible for this program. According to the recent census (2016), 31.16% of Iranians were older than 40 years (6). By adding individuals aged older than 30 years with the aforementioned risk factors, we can conclude that this program is going to screen at least 25 million people yearly.

The healthcare perspective and a 40-year time horizon were adopted for this analysis. As the analysis is a comparison between IraPEN (intervention) and *status quo* (no intervention) which both have the same Markov structure and transition probabilities, it is not expected that half cycle correction (HCC) approach makes any difference in ICER results; therefore, HCC was not applied to this analysis (7).

The hypothetical cohorts were used as a representative for individuals with low, moderate, high, and very high CVD risk profiles (Table 1). Progressively, a proportion of the cohort can go to the CHD state, who are the survivors of the first CHD event, or to the stroke state who are the survivors of the first stroke event. Those CHD and stroke events that were fatal moved to the death state. In general, the people in these two states are at a higher risk of dying from CHD or stroke, but they may die from any other causes like the normal population. Table 2 summarizes the assumptions of this analysis.

**Table 1:** Index cohorts representing different CVD risk levels^*^.

	**Low risk**	**Moderate risk**	**High risk**	**Very high risk**
**Without diabetes**				
Systolic blood pressure	120–139 mmHg	140–159 mmHg	160–179 mmHg	>180 mmHg
Total cholesterol	< 195 mg/dl	>310 mg/dl	>310 mg/dl	>310 mg/dl
HDL	40 mg/dl	46 mg/dl	46 mg/dl	41 mg/dl
Smoking	No	Yes	Yes	Yes
Sex	Male	Female	Female	Male
**With diabetes**				
Systolic blood pressure	120–139 mmHg	140–159 mmHg	160–179 mmHg	>180 mmHg
Total cholesterol	<195 mg/dl	>310 mg/dl	<270 mg/dl	<270 mg/dl
HDL	46 mg/dl	46 mg/dl	40 mg/dl	41 mg/dl
Smoking	No	No	Yes	Yes
Sex	Female	Female	Male	Male

* Green color: represents the low-risk group (individuals with CVD risk < 10%). Yellow color: represents the moderate-risk group (individuals with CVD risk between 10 and 19%). Orange color: represents the high-risk group (individuals with CVD risk between 20 and 29%). Red color: represents the very high-risk group (individuals with CVD risk more than 30%).

**Table 2:** Main elements of this economic evaluation (EE).

Comparators	IraPEN intervention vs. status quo (no prevention)
Perspective	Healthcare
Target group	All Iranians older than 40 years old
Type of EE	Cost-effectiveness analysis by adopting a Markov model
Considered costs	All direct medical costs
Discount rate	3.5% for costs and effects
Sensitivity analysis	Determinstic and Probabilistic sensitivity analysis (DSA and PSA)
CE threshold	GDP per capita of Iranian people—$4,091

### Data input

This analysis tried to use the Iranian data wherever available. In case of a lack of local data, the inputs were derived from the global literature. Therefore, all transition probabilities of the models were extracted from available Iranian data, while medications' effects and states' utilities were driven from the literature of Western countries (refer to Supplementary material). No individual data have been used for this analysis.

### Transition probabilities

The annual incidence rate for CHD (8) and stroke was calculated from the Framingham study equations (9). As four-index cohorts had been defined with specific characteristics, there was a need to calculate the risk based on those profiles. Based on the literature, one out of four CHD events are fatal in the first year (10), while 60% of them are pre-hospital deaths (11). Therefore, it was assumed that of those who have a CHD event in the model, 25% die in the first year. Approximately 60% of these deaths were costless as they are pre-hospital deaths. Regarding first-year stroke mortality, the range varies in different resources and is reported from 22 to 34% (12). For this analysis, the rate was applied from the largest available cohort (13). Almost 25% of stroke events are fatal in the first year, while half of them occur during the first 28 days. Therefore, it is assumed that although at the end of the cycle, they move to the death state, 40% of the cycle cost should be considered for them. The fatality rate for stroke and CHD survivors was derived from a study that had been done on the Iranian population (14). The background mortality rate from all causes other than stroke and CHD is calculated by excluding the total death attributed to these two diseases from the Iranian life table^1^. The total mortality rate of these two events had been calculated in Tehran Lipid and Glucose Study (TLGS). At first, the annual rates were derived from the life table and then the CHD- and stroke-attributed deaths were excluded.

### Intervention effect

The IraPEN's preventive actions are expected to reduce cardiovascular events. The relative risks (RRs) of these preventive actions and the medications that are used in the program were obtained from meta-analyses or randomized clinical trials (RCTs). By multiplying or adding up the RRs of different medications, there is a risk of effect overestimation, and a correction was made by using the formula below wherever multiple interventions were involved:


$$\begin{array}{l} 1-\;\left(\left(1-\;{\text{RR}}_{1}\;\times \;{\text{RR}}_{2}\;\times \;{\text{RR}}_{\text{N}}\right)\times \;0.8\right). \end{array}$$

This equation has been developed based on a study that compared the effect of controlling the risk factors separately vs. controlling all of them simultaneously (15).

Based on the field interviews, it was clear which medications are used for each index cohort. Almost in all cases, angiotensin-converting enzyme (ACE) inhibitors are the first choice for hypertension treatment. Enalapril is the most prescribed one as monotherapy. Thiazides (diuretics) are the second choice followed by beta-blockers. In case the hypertension is not controlled by monotherapy instead of increasing the dose, the second drug is added. As recommended by guidelines, small doses of various classes of antihypertensive medications are more useful than a high dose of one (16). In general, the combination of ACE inhibitors and thiazide is the most common one. This pattern is aligned with Joint National Committee (JNC8) guidelines. Statins are prescribed for hyperlipidemia treatment. Among statins, Atorvastatin is the choice as it is one of the most potent ones. For diabetes, Metformin is started and increased to the maximum dose (2 g) and then the second medication that is Glibenclamide is added. Due to its potential harm and insufficient evidence of its efficacy, Aspirin was not recommended for primary prevention by PEN protocols. Therefore, Aspirin is not used in IraPEN as well. Here are the list of medications and their daily dosages which are used in IraPEN:

- Atorvastatin 20 mg tablet for statin therapy (statin).
- Enalapril Maleate 20 mg tablet is the first choice for hypertension treatment (Ace inhibitor).
- Hydrochlorothiazide 50 mg tablet (diuretics second choice).
- Metoprolol tartrate 50 mg tablet (beta-blocker third choice).
- Metformin HCL 500 mg tablet for diabetes (daily consumption from 500 to 2 g).
- Glibenclamide 5 mg tablet is the second choice for diabetes.

The unit price of each of these medications was derived from the Iranian Annual Pharma Statistics file. For the calculation of the intervention's effects, it is assumed that the adherence of individuals to the treatment is 100%. Table 3 lists the RRs of different interventions (medications) for CHD and stroke.

**Table 3:** Intervention effects based on the subclass of medications that are used in IraPEN.

		**RR**	**(95% CI)**
**Angiotensin-converting enzyme inhibitor** **(****17****)**			
RR	CHD	0.81	(0.70–0.94)
	Stroke	0.65	(0.52–0.82)
**Thiazide diuretics** **(****18****)**			
RR	CHD	0.84	(0.75–0.95)
	Stroke	0.63	(0.57–0.71)
**Beta-blockers** **(****19****)**			
RR	CHD	0.90	(0.78–1.03)
	Stroke	0.83	(0.72–0.97)
**Statin** **(****20****)**			
RR	CHD	0.86	(0.82–0.90)
	Stroke	0.90	(0.85–0.95)
**Metformin** **(****21****)**			
RR	CHD	0.67	(0.51–0.89)
	Stroke	0.80	(0.50–1.27)
**Sulfonylureas**			
RR	CHD	0.85	(0.74–0.97)
	Stroke	0.91	(0.73–1.13)
**Lifestyle counseling** **(****22****)**			
RR	CHD	0.86	(0.81–0.91)
	Stroke	0.86	(0.81–0.91)

### Costs

A healthcare perspective was adopted; therefore, we only included costs associated with healthcare such as direct medical costs (Table 4). The costs considered in the model are the cost of IraPEN screening, the cost of IraPEN monitoring, the cost of CHD survivors, and the cost of stroke survivors. It is assumed that the cost of individuals who are event-free in the *status quo* is zero as long as undiagnosed or untreated. These two facts were considered for the *status quo* costs. Furthermore, it was assumed that the cost of dying was equal to zero. According to PEN protocols, the needed resources for each index cohort were identified. Then, the items were quantified based on discussions with the physicians and supervisors of the visited centers. The cost of index cohorts consists of two different types. First, variable costs are different for each group based on the characteristics of each. Second, fixed cost is the same for all and consists of staff training, administration, IT, promotional stuff, and leaflets. The unit price of each item was derived from the last report of the Ministry of health (23). The report estimated all the costs related to IraPEN implementation except the medications. In addition, the reported costs were adjusted by the 2018 inflation rate and the cost of each cohort was calculated.

**Table 4:** Annual cost of each index cohort with diabetes.

	**Low-risk**	**Moderate-risk**	**High-risk**	**Very high-risk**
Behvarz's visit (Screening)	108,173	324,519	432,692	432,692
Physician visit	–	216,346	288,462	432,692
Lab data (included in screening)		–	–	–
Nutrition consultation	38,400	38,400	38,400	38,400
Psychiatrist consultation	–	–	38,400	38,400
Anti-hypertensive medication	–	One agent	Two agents	Three agents
Statin Therapy	–	+	+	+
Fixed costs	19,231	19,231	19,231	19,231
Cost of each group in 2017 (IRR)	165,804	598,496	817,185	961,416
The inflation rate was applied to the costs (IRR)	216,540	781,636	1,067,243	1,255,609
Cost of each index cohort without medication	$5.16	$18.61	$25.41	$29.90
Cost of medication of each index cohort	$16	$38.85	$48.27	$55.39
**Cost of each index cohort for the model**	**$21.63**	**$57.46**	**$73.68**	**$85.29**

Exchange rate: 42,000 IRR = 1 US dollar. Bold indicates total cost of each index cohort for the model.

Green color: represents the low-risk group (individuals with CVD risk less than 10%); Yellow color: represents the moderate-risk group (individuals with CVD risk between 10% to 19%); Orange color: represents the high-risk group (individuals with CVD risk between 20% to 29%); Red color: represents the very high-risk group (individuals with CVD risk more than 30%).

The cost of CHD state and stroke state was derived from an Iranian CE that had estimated the cost of these two states (24). These two costs contain all the related medical costs such as hospital admissions and procedures, monitoring, follow-ups, medications, and secondary prevention (Table 5). Based on experts' opinions, it is assumed that the cost of CHD after the first year would be a third and the cost of stroke state after the first year would be a quarter. In addition, it is assumed that the standard error of costs for the consecutive year is 10% of the mean.

**Table 5:** Annual cost of CHD and stroke states.

	**Mean**	**SE**
CHD cost for the first year	$519	$51
CHD cost for consecutive years	$173	$17
Stroke cost for the first year	$5,691	$569
Stroke cost for consecutive years	$1,422	$142

### Utilities

As all people who enter the model are healthy individuals, the utility for the first health state is considered 1. For the death health state utility, it was adopted the standard approach by setting the utility to zero. CHD and stroke state utilities were derived from the published literature. In the models after the first event, patients move to these states and stay there until they die. Although they remain in the same states (CHD or stroke), their utilities are different over time. From a medical view, acute post-event utilities are (much)lower than chronic post-event utilities. Therefore, it was essential to use the data from the study which assessed the acute and chronic utilities with the same participants at an appropriate time. For this purpose, the utilities were derived from the study which assessed the utilities within the first year and consecutive years (13). Table 6 shows the utilities used for the model. All the costs and effects were discounted at the rate of 3.5%.

**Table 6:** Utility weights for CHD and stroke states.

	**Utility mean**	**SE**
Utility of CHD survivors—first year	0.67	0.024
Utility of stroke survivors—first year	0.33	0.033
Utility of CHD survivors—second year onwards	0.82	0.012
Utility of stoke survivors—second year onwards	0.52	0.027

### Sensitivity analysis

To quantify the level of confidence in the models' results, a deterministic sensitivity analysis (DSA) and a probabilistic sensitivity analysis (PSA) were performed. In the DSA, the input parameters were varied to the maximum and minimum possible values. This range is usually defined by the confidence interval of parameters. Therefore, for the examined parameters, a range of 95% confidence interval was specified and then based on this range (maximum and minimum input values), one value in the model was varied manually each time. For the patients' adherence, the range of 50%−100% was considered. The results (new ICERs) were collected and expressed with tornado diagrams. The tornado diagrams depict the impact on the ICER whenever one single parameter changed.

For the PSA, as the main assumption, it was considered the deterministic input values in the parameter sheet as the mean values. As the standard errors of the cost items were not available, it was considered to mean value times by 0.1. Based on logical constraints, the probabilistic distribution for each of the different sources of uncertainty was defined. A gamma distribution for all cost items and a beta distribution for the utilities were defined. The PSA was conducted by drawing a random number for each of the input distributions and each time, the ICER was calculated by Excel. By running a macro, its action is repeated 1,000 times.

## Results

For the interpretation of the results, 1 GDP per capita was assumed as the CE threshold, which is equal to $4,091 (25). In Table 7, the results of the “CVD risk model without diabetes” are reported. The IraPEN intervention for individuals having moderate CVD risk yields an ICER of $804 per QALY, while for high-risk groups, this intervention provides an ICER of $551 per QALY. The results showed that this intervention would be cost-saving and improve health if the IraPEN program targets only individuals with a CVD risk higher than 30%. The model yields the ICER of –$44 per QALY for this group. Moreover, the model results showed that individuals with higher CVD risks gained higher LY out of the intervention. The moderate, high, and very high CVD risk groups gained 0.69, 0.96, and 1.45 LY, respectively, from the intervention. Table 8 shows the model results for the “CVD risk model with diabetes”.

**Table 7:** Base-case results for CVD risk groups without diabetes.

	**COST**	**QALY**	**LY**	**ICER**
Status quo	$845	18.12	32.27	
IraPEN	$918	18.12	32.27	Undefined
Incremental	$73	0	0	Low-risk
Status quo	$979	17.89	32.37	
IraPEN	$1,375	18.38	33.06	$804 ♀
Incremental	$396	0.49	0.69	Intermediate-risk
Status quo	$1,204	17.68	32.13	
IraPEN	$1,594	18.38	33.09	$551 ♀
Incremental	$391	0.71	0.96	High-risk
Status quo	$2,348	15.83	29.3	
IraPEN	$2,296	17.02	30.75	–$44 ♂
Incremental	–$52	1.19	1.45	Very high-risk

CE threshold = $4,091, ♂ = male, ♀ = female.

Green color: represents the low-risk group (individuals with CVD risk less than 10%); Yellow color: represents the moderate-risk group (individuals with CVD risk between 10% to 19%); Orange color: represents the high-risk group (individuals with CVD risk between 20% to 29%); Red color: represents the very high-risk group (individuals with CVD risk more than 30%).

**Table 8:** Base-case results for CVD risk groups with diabetes.

	**COST**	**QALY**	**LY**	**ICER**
Status quo	$647	18.59	33.36	Low risk
IraPEN	$866	18.9	33.82	$711 ♀
Incremental	$219	0.31	0.46	
Status quo	$1,027	17.75	32.17	Moderate
IraPEN	$1,560	18.59	33.37	$630 ♀
Incremental	$533	0.85	1.2	
Status quo	$2,429	15.91	29.46	High
IraPEN	$2,361	17.53	31.5	–$42 ♂
Incremental	–$68	1.61	2.04	
Status quo	$2,810	15.31	28.74	Very high
IraPEN	$2,678	17.18	31.03	–$71 ♂
Incremental	–$133	1.87	2.29	

CE threshold = $4,091, ♂ = male, ♀ = female.

Green color: represents the low-risk group (individuals with CVD risk less than 10%); Yellow color: represents the moderate-risk group (individuals with CVD risk between 10% to 19%); Orange color: represents the high-risk group (individuals with CVD risk between 20% to 29%); Red color: represents the very high-risk group (individuals with CVD risk more than 30%).

Here, the results demonstrate that the intervention would be cost-saving while improving health if target individuals with CVD risk comprise higher than 20%. The intervention yields an ICER of –$42 per QALY for the high-risk group and an ICER of –$71 per QALY for the very high-risk group. Similar to the previous model, individuals with higher risks gain more LY from the intervention.

**Figure 2:**
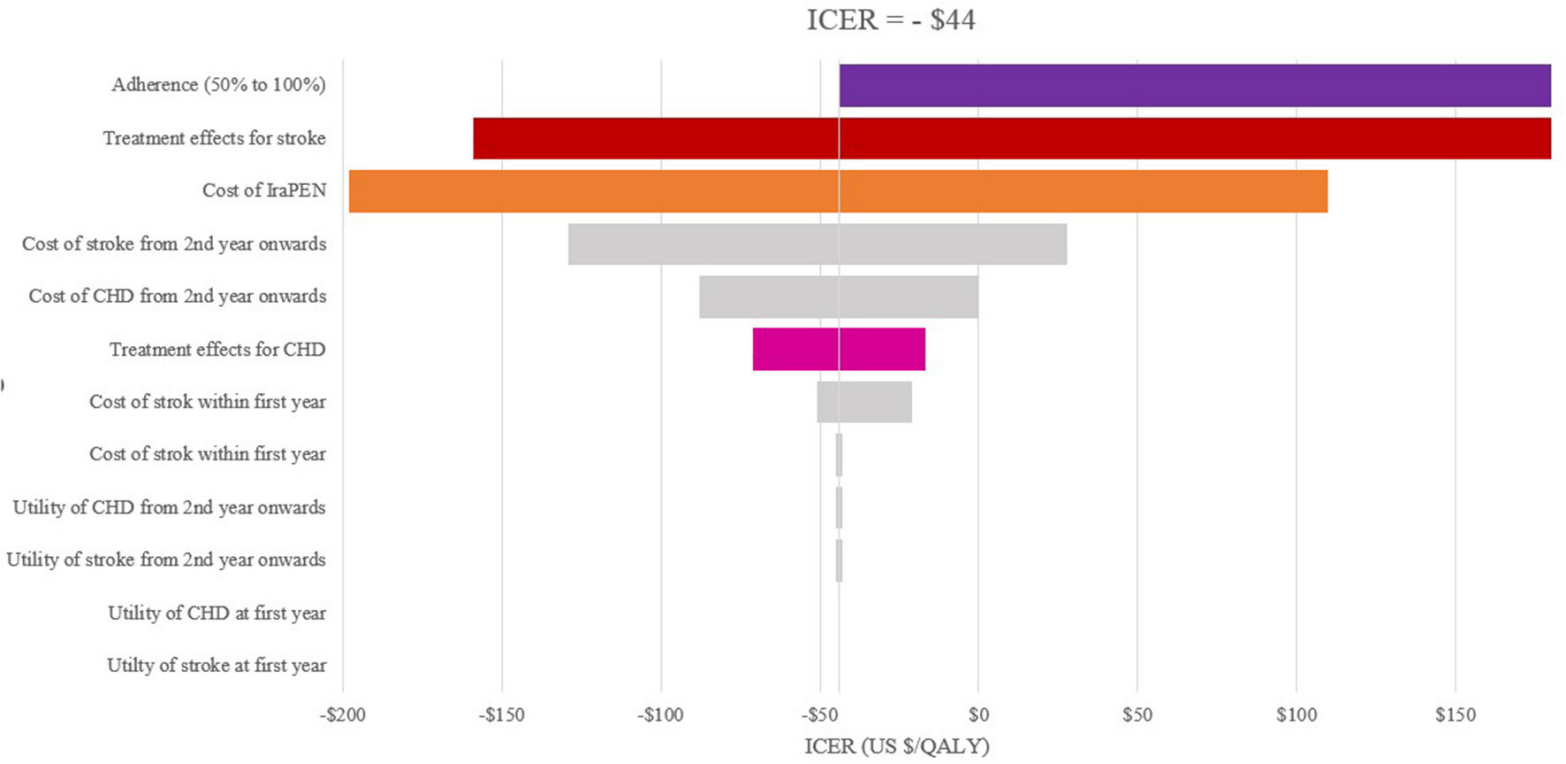
One-way sensitivity analysis of modeled cost–effectiveness of CVD risk-based prevention for very high-risk without diabetes group.

**Figure 3:**
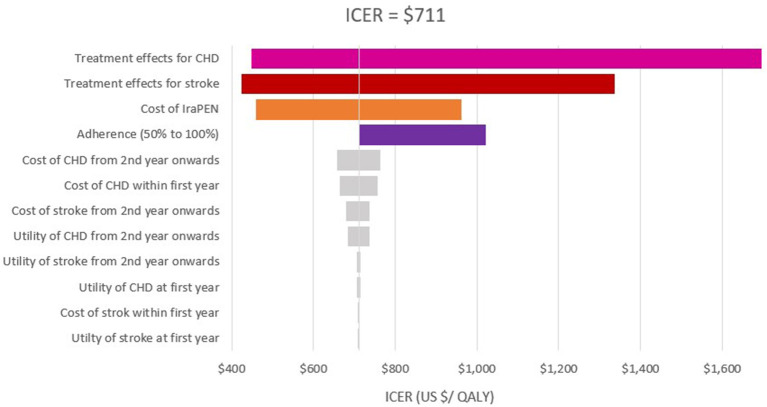
One-way sensitivity analysis of modeled cost–effectiveness of CVD risk-based prevention for low risk with diabetes group.

The one-way sensitivity analysis reveals that the patients' adherence, the treatment effectiveness, and the total cost of the IraPEN program have the most impact on the ICER. The influence of patients' adherence is more noticeable in the higher CVD risk groups, while the results are more sensitive to treatment effectiveness in the lower risk groups (Figures 2, 3).

In the scenario analysis of 50% adherence, an ICER of $1,451, $1,141, and $329 was achieved for moderate, high, and very high CVD risk in groups without diabetes, respectively. In this scenario, the intervention yields an ICER of $1,029, $1,022, $236, and $199 for low, moderate, high, and very high CVD risk groups with diabetes, respectively. It was found that the ICERs are lower than the threshold at both the upper and lower limits of all examined parameters.

**Figure 4:**
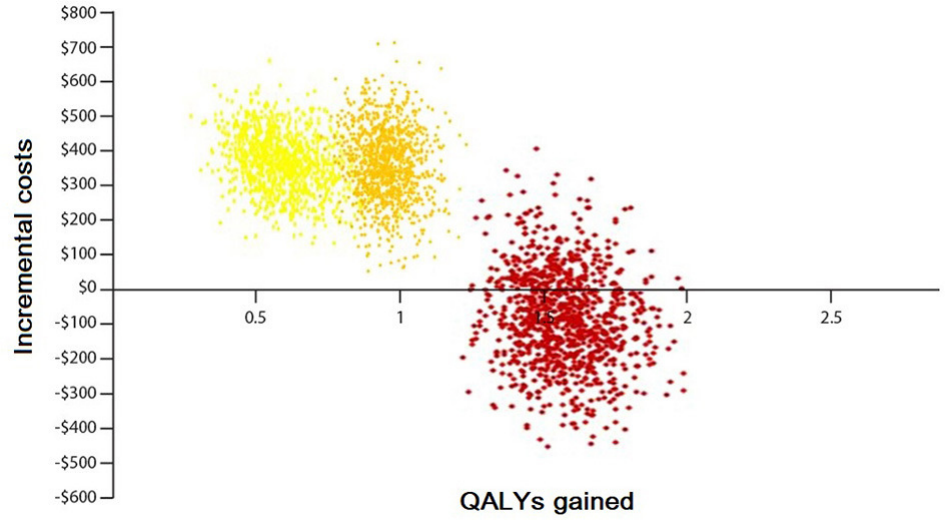
Probabilistic sensitivity analysis of the Markov model without diabetes. Yellow color: represents the moderate-risk group (individuals with CVD risk between 10 and 19%). Orange color: represents the high-risk group (individuals with CVD risk between 20 and 29%). Red color: represents the very high-risk group (individuals with CVD risk more than 30%).

**Figure 5:**
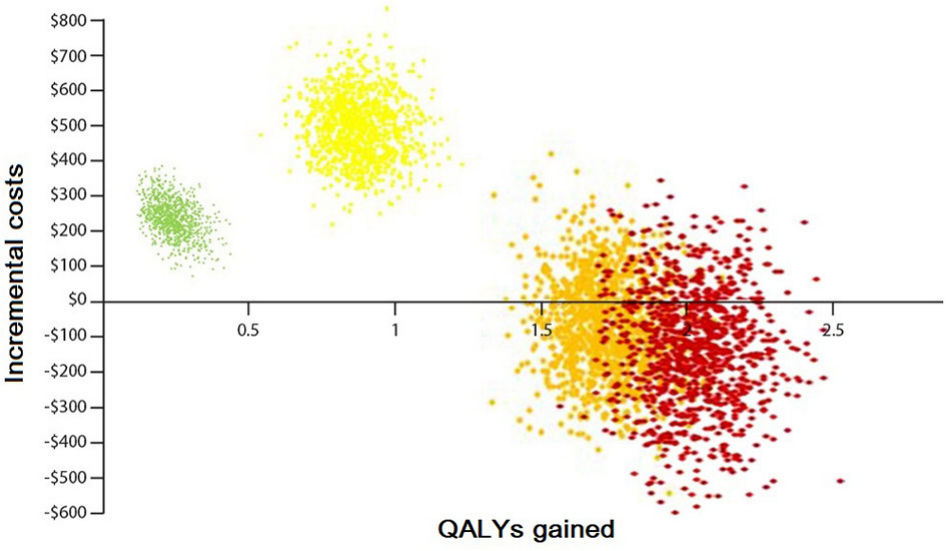
Probabilistic sensitivity analysis of the Markov model with diabetes. Green color: represents the low-risk group (individuals with CVD risk <10%). Yellow color: represents the moderate-risk group (individuals with CVD risk between 10 and 19%). Orange color: represents the high-risk group (individuals with CVD risk between 20 and 29%). Red color: represents the very high-risk group (individuals with CVD risk more than 30%).

The result of 1,000 PSAs illustrates that the intervention for all groups, except the low CVD risk group without diabetes, is cost-effective while being cost-saving for at least half of high-risk and very high-risk patients (Figures 4, 5). By adopting 1 GDP per capita of Iran as the willingness to pay per quality-adjusted life-year (WTP/QALY) gained, 100% of all the runs were cost-effective in these groups.

Both models captured that men would be benefited more than women in terms of LY gained. In addition, the intervention generates a lower ICER for men than women in individuals with identical characteristics. For example, in the low CVD risk group with diabetes, the interventions yield an ICER of $239 per QALY for men, while it is $711 per QALY for women. Regarding the LY in this group, in equal circumstances, men saved 0.58 of a year and women saved 0.46 of a year. In higher risk groups, this difference is more prominent. For example, for very high-risk groups, regardless of their diabetes status, the intervention for men is cost-saving, while for women it is not (Tables 9, 10).

**Table 9:** Heterogeneity base-case results for very high CVD risk group with diabetes.

	**COST**	**QALY**	**LY**		**ICER**
Status Que	$2,810	15.31	28.74	♂	
IraPEN	$2,678	17.18	31.03	♂	–$71
Incremental	-$133	1.87	2.29	♂	
Status Que	$2,133	16.17	30.18	♀	
IraPEN	$2,393	17.9	32.42	♀	$151
Incremental	260	1.73	2.24	♀	

CE threshold = $4,091, ♂ = male, ♀ = female.

Red color: represents the very high-risk group (individuals with CVD risk more than 30%).

**Table 10:** Heterogeneity base-case results for very high CVD risk group without diabetes.

	**COST**	**QALY**	**LY**		**ICER**
Status Que	$2,348	15.83	29.3	♂	
IraPEN	$2,296	17.02	30.75	♂	−44
Incremental	-$52	1.19	1.45	♂	
Status Que	$1,522	17.19	31.46	♀	
IraPEN	$1,912	18.13	32.71	♀	418
Incremental	390	0.93	1.25	♀	

CE threshold = $4,091, ♂ = male, ♀ = female.

Red color: represents the very high-risk group (individuals with CVD risk more than 30%).

## Discussion

This analysis aimed to measure the potential CE of IraPEN preventive actions for CVD in comparison with the *status quo*. Our results illustrated that this intervention is not cost-effective for the low CVD risk group, whereas the other groups under study proved to be highly cost-effective. The reason why this group was not cost-effective could be justified by the fact that the low CVD risk group has lower CVD risk factors, which is why such individuals are just screened without being intervened.

In this study, we adopted the CE threshold, i.e., 1–3 GDP per capita, as proposed by WHO (26). The study of the literature shows that by using this threshold, almost all interventions seem to be cost-effective (27). It means that by adopting this CE threshold, there is a risk that the budgets are spent on interventions that should not and vice versa. The threshold which is recommended by WHO has received some criticism as it is believed that it does not reflect the true “opportunity cost.” This is more critical in low- to middle-income countries, because while they have a higher demand for health, in comparison with high-income countries, fewer resources are available to them. In 2016, Woods et al. (28) calculated the CE threshold based on the empirical estimates of opportunity cost, the relationship between a country's GDP per capita, and the value of statistical life. For that reason, they estimated the threshold for different countries with different levels of income. Based on their estimation, Woods et al. suggested the CE threshold to be about 50% of GDP per capita. As appointing the precise CE threshold level is beyond the scope of our analysis, we interpret and discuss the results with the lowest recommended ICER and leave the decision to policymakers to choose an intervention that best fits their budget. The latest reported GDP per capita of Iran is $4,091. By comparing the results with this threshold, it is shown that all of them, except the low CVD risk group, are highly cost-effective, whereas if the program only targets the people in higher risk groups, it is both cost-saving and improves their health.

Based on the results, the intervention for the low-risk group was not cost-effective as the ICER was undefined. This could be explained by the fact that in this group, just screening is done without offering any intervention. So here, there is a cost for screening without any tangible effect assigned. Since this group does not receive any treatment annually, it is, by all means, sensible that no effects are observed. While it seems to be justifiable, we have every reason to believe that this is the only way to find the groups with a higher CVD risk. Another point that should be mentioned is that the individuals enter and are screened in our model at the age of 40 years. At this age, the proportion of individuals with low CVD risk is significantly higher than that of those with higher CVD risk. The older a person becomes, the more the probability of being in the higher-risk groups will be.

However, the fact that the intervention for the aforementioned group is not cost-effective needs to be approached more comprehensively and conservatively. Screening of this group is the first step and essential for all individuals in the other groups that cannot be disregarded. This means that the other groups can benefit from this, a fact that has not been considered in the analysis of the CE of this group in this study.

From another perspective, a closer observation reveals that screening has a wide range of benefits. For instance, according to the latest national data (29), the prevalence of people with diabetes in Iran is 11.4%, a quarter of whom are undiagnosed (30). This means that at the moment, there are almost 1.5 Iranian people with undiagnosed diabetes. These undiagnosed individuals are discovered only when their complications have started to appear in them. Such complications as retinopathy, nephropathy, and neuropathy are very costly and can impose burdensome pressures on the healthcare system of the country. Other typical examples of this kind are blood hypertension and hyperlipidemia.

It is predicted that huge monetary resources should be allocated to the overall screening of all the individuals, which may not be conveniently supplied. Therefore, appropriate measures could be taken by the authorities to have the costs tailored. This can help manage the financial resources and distribute them as optimally as possible. If the available financial resources do not allow us to screen everyone, it is possible to screen all high-risk people. Although this may not sound optimal, still it has a lot of benefits to offer. In other words, when considered at a higher scale, it can be realized that since the proportion of high CVD risk group individuals outweighs those with lower risks, this may lead to much more favorable results.

It is applicable to have a paper pre-screening. The idea is that alternatively, paper questionnaires can be distributed among both households and health center visitors. These questionnaires aim to detect individuals with higher CVD risks. Typical examples might be those who are obese or have a positive history of CVDs in their intimidate relatives. Once identified, such participants can be invited to health centers to get screened. In this way, we can narrow the target population and screen those who are at higher risk levels.

The study of the literature suggested some good examples of this kind of practice. For example, Chamnan et al. (31), who conducted a modeling study using the data from a prospective cohort study (EPIC-Norfolk), concluded that adopting a stepwise screening approach can prevent the same number of CVD events annually. All the participants of the EPIC-Norfolk study had completed the questionnaire about their lifestyle and drug use and family history of diseases between 1993 and 1997. This population had been observed and followed for 10 years and all CVD events had been recorded. By adopting the Cambridge risk score (a British risk scoring tool) and based on the results of completed questionnaires, the Chamnan group ranked the population CVD risk. Then, they defined and modeled seven different stepwise screening strategies. By comparing the results of a 10-year follow-up of the population with their model, they found that inviting the individuals with a Cambridge risk score >60 might have had the same results as screening the population. According to their report, this strategy could have enabled them to have the same results about the whole population by screening only 60% of the population. Similar results were observed by Móczár and Rurik (32) who concluded that performing screening for a selected target group is most likely to be more cost-effective than screening the whole population. It is essential to consider that although this approach is practical and likely more cost-effective in budget-strained situations, it has some serious ethical issues regarding equity because in this approach, a smoker would get screened and a non-smoker would not. The same is true for a person who has an unhealthy diet or lifestyle. Moreover, while it is conveniently applicable to rural areas, it is not easy to do in urban areas.

Since our literature search revealed no similar studies either in the WHO East Mediterranean region (EMR) or in the Middle East region, inevitably, we compared the results of our analysis with the European studies. Our findings in this analysis, in terms of CE and the trend between the different CVD risks, are similar to the results of Schuetz et al.'s (33) study. The researchers of this study estimated the CE of several different preventive strategies compared with a control scenario in six European countries. By using country-specific data from France, Germany, Denmark, Italy, Poland, and the United Kingdom, they generated six simulated populations of people aged 40–75 years eligible for preventive actions in those countries. Their model showed that the cost per QALY of offering these preventive services to the people in the study cohort ranged from €14,903 for France to €115 for Germany, while it was cost-saving for Poland. Their results showed that the health checks for detecting and managing CVDs at the early stages not only are highly cost-effective but also cost-saving in some scenarios. For example, their analysis for the UK showed that during the 30-year follow-up, the cost per QALY would be €2,426. This ICER in comparison with the UK threshold, which is between 20 and 30 thousand pounds, is highly cost-effective. Moreover, their results demonstrated that the program would be cost-saving if it targets only the top quartile of CVD risk groups. Furthermore, offering prevention checks after the pre-screening of individuals based on some characteristics such as higher age or obesity would pull the results in the direction of more favorable ICER (33).

Finally, while this screening program can have substantial benefits for individuals with CVD risk, it is essential to consider the potential disutility of lifelong preventive treatments. The search of the literature indicates that the disutility of medications' adverse events (34) and the disutility arising from taking daily medication (35) can play a key role in the decision that leads to non-adherence. It is vital to take into account that some individuals need to be on preventive treatments from their 40s for around 30–40 years. Although the sensitivity analysis demonstrated that this program can likely be cost-effective even with 50% of adherence, it is crucial to enhance treatment compliance through patient education and take effective strategies to increase the engagement of target groups.

### Strengths and limitations

Based on our literature search, this analysis is the first CVD risk-based CE to be conducted in Iran, in the Middle East, and the WHO EMR so far. Furthermore, it is one of the few studies which model the individual with and without diabetes separately.

Similar to every CE analysis, this study has several limitations which were mainly caused by a large number of input parameters used in the model. Although the model was designed for the Iranian population, some input parameters were derived from various studies performed in different countries other than Iran directly because of the unavailability or a lack of Iranian data. Furthermore, it was assumed that the intervention effect is equal for all subgroups regardless of their initial CVD risk. Such an assumption can probably generate an underestimation of the intervention effect for high-risk groups and produce an overestimation of the treatment effect for lower CVD risk groups.

In this analysis, the second event of CVDs was not accounted for due to a lack of data. Based on the literature, almost 50% of patients would experience the second or third event during their life once they have had the first event (36). The second event may not necessarily be the same as the first one. For example, a patient who has had a CHD event could have the same event again or can even experience a stroke event. This cannot affect the result of our analysis unfavorably. As the IraPEN intervention causes the first CVD event to decrease or delay, it is logical to assume that the second event in the IraPEN group is lower than the *status quo*. Therefore, we could assume that adding the second CVD event to our model would be in favor of our ICER.

It could be better if we could adopt a lifetime horizon for this analysis. However, as the Iranian statistical data were just available for people aged below 80 years, inevitably 40 years/cycles were employed. The results of Kim et al.'s (37) systematic review, which was done on more than 750 CE analyses, showed that the usage of a lifetime horizon captures all consequences and health benefits most of the time and yields more favorable ICER. Therefore, it is logical to assume that by increasing the time horizon from 40 years to a lifetime, the ICER would be more favorable.

Finally, it should be expected that the effectiveness of the intervention would be lower in the real world than in the model. It could be explained by the fact that the intervention effects, which were used in this study, all had been derived/extracted from a controlled trial setting.

## Conclusion

In Iran, CVDs are the leading cause of mortality. Therefore, planning and implementing preventive actions are highly demanded. Our analysis results demonstrated that the IraPEN program implementation is highly cost-effective for all the CVD risk groups, except the low risk without diabetes group, whereas if the program only targets the people in higher risk groups, it is both cost-saving and improves their health.

## Data availability statement

The original contributions presented in the study are included in the article/Supplementary material, further inquiries can be directed to the corresponding author/s.

## Author contributions

AJ contributed to the design, carried out the analysis, and wrote the first draft. RD contributed to the analysis and reviewed the manuscript critically. EA contributed to the design and supervised the process of analysis reviewed the manuscript critically. DK designed the whole project and contributed to the analysis and drafting of the manuscript. All authors read the final manuscript and approved it.

## Footnotes

^1^ https://www.who.int/data/gho/data/themes/topics/indicator-groups/indicator-group-details/GHO/gho-ghe-global-health-estimates-life-tables

## References

[1] Chrysant S. A new paradigm in the treatment of the cardiovascular disease continuum: focus on prevention. Hippokratia. (2011) 15:7–11. 21607028 PMC3093150

[2] Azizi F Hadaegh F Hosseinpanah F Mirmiran P Amouzegar A Abdi H. Metabolic health in the Middle East and north Africa. Lancet Diabetes Endocrinol. (2019) 7:866–79. 10.1016/S2213-8587(19)30179-2 31422063

[3] Azadnajafabad S Mohammadi E Aminorroaya A Fattahi N Rezaei S Haghshenas R. Non-communicable diseases' risk factors in Iran; a review of the present status and action plans. J Diabetes Metab Disord. (2021) 22:1–9. 10.1007/s40200-020-00709-8 33500879 PMC7821170

[4] Hadavand Siri F Khalili D Hashemi Nazari SS Ostovar A Mahdavi A. Adherence to Iran's package of essential noncommunicable diseases (IraPEN) program for regular follow-up to reduce the risk of cardiovascular disease in healthcare centers. Iran J Endocrinol Metab. (2020) 22:116–26.

[5] World Health Organization. WHO/ISH Cardiovascular Risk Prediction Charts. Geneva: World Health Organization (2011). Available online at: https://www.who.int/cardiovascular_diseases/guidelines/Chart_predictions/en/ (accessed February 20, 2019).

[6] 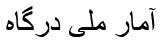 . Available online at: https://www.amar.org.ir/ (accessed June 10, 2019) .

[7] Naimark DMJ Kabboul NN Krahn MD. The half-cycle correction revisited. Med Decis Making. (2013) 33:961–70. 10.1177/0272989X13501558 24048350

[8] Andreson K Wilson P Odell P Kannel W. An updated coronary risk profile a statement for health professionals. Circulation. (1991) 83:356–62. 10.1161/01.CIR.83.1.356 1984895

[9] Wolf P D'agostino R Belanger A Kannel W. Probability of stroke: a risk profile from the Framingham Study. Stroke. (1991) 22:312–8. 10.1161/01.STR.22.3.312 2003301

[10] Talaei M Sarrafzadegan N Sadeghi M Oveisgharan S Marshall T Thomas GN. Incidence of cardiovascular diseases in an Iranian population: the Isfahan Cohort Study. Arch Iran Med. (2013) 16:138–44. 23432164

[11] Grey C Jackson R Schmidt M Ezzati M Asaria P Exeter DJ. One in four major ischaemic heart disease events are fatal and 60% are pre-hospital deaths: a national data-linkage study (ANZACS-QI 8). Eur Heart J. (2015) 38:172–80. 10.1093/eurheartj/ehv524 28158544

[12] Shoeibi A Salehi M Thrift AG Kapral MK Farzadfard MT Azarpazhooh A. One-year case fatality rate following stroke in the Mashhad Stroke Incidence Study: a population-based study of stroke in Iran. Int J Stroke. (2015) 10(Suppl A):96–102. 10.1111/ijs.12611 26502970

[13] Matza LS Stewart KD Gandra SR Delio PR Fenster BE Davies EW. Acute and chronic impact of cardiovascular events on health state utilities. BMC Health Serv Res. (2015) 15:173. 10.1186/s12913-015-0772-9 25896804 PMC4408571

[14] Rabani SH Sardarinia M Akbarpour S Azizi F Khalili D Hadaegh F. 12-year trends in cardiovascular risk factors (2002-2005 through 2011-2014) in patients with cardiovascular diseases: Tehran lipid and glucose study. PLoS ONE. (2018) 13:e0195543. 10.1371/journal.pone.0195543 29768511 PMC5955533

[15] Dagenais GR Pogue J Fox K Simoons ML Yusuf S. Angiotensin-converting-enzyme inhibitors in stable vascular disease without left ventricular systolic dysfunction or heart failure: a combined analysis of three trials. Lancet. (2006) 368:581–8. 10.1016/S0140-6736(06)69201-5 16905022

[16] Xavier D Noby M Pradeep J Prem P. Letter to the editor. Pattern of drug use in hypertension in a tertiary hospital; a cross sectional study in the inpatients ward. Indian J Pharmacol. (2001) 33:456–7.

[17] Wright J Musini V. First-line drugs for hypertension. São Paulo Med J. (2010) 128:47. 10.1590/S1516-31802010000100011

[18] Wright JM Musini VM Gill R. First-line drugs for hypertension. Cochrane Database Syst Rev. (2018) 4:CD001841. 10.1002/14651858.CD001841.pub3 29667175 PMC6513559

[19] Law MR Morris JK Wald NJ. Use of blood pressure lowering drugs in the prevention of cardiovascular disease: meta-analysis of 147 randomised trials in the context of expectations from prospective epidemiological studies. Br Med J. (2009) 338:b1665. 10.1136/bmj.b1665 19454737 PMC2684577

[20] Brugts J Yetgin T Hoeks S Gotto A Shepherd J Westendorp R. The benefits of statins in people without established cardiovascular disease but with cardiovascular risk factors: meta-analysis of randomised controlled trials. BMJ. (2009) 338:b2376. 10.1136/bmj.b2376 19567909 PMC2714690

[21] Holman RR Paul SK Bethel MA Matthews DR Neil HAW. 10-year follow-up of intensive glucose control in type 2 diabetes. N Engl J Med. (2008) 359:1577–89. 10.1056/NEJMoa0806470 18784090

[22] Lanier J Bury D Richardson S. Diet and physical activity for cardiovascular disease prevention. Am Fam Physician. (2016) 93:919–24. 27281836

[23] 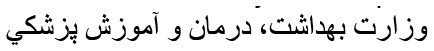 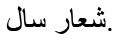 Available online at: http://behdasht.gov.ir/ (accessed March 29, 2019).

[24] Javanbakht M Jamshidi AR Baradaran HR. Estimation and prediction of avoidable health care costs of cardiovascular diseases and type 2 diabetes through adequate dairy food consumption: a systematic review and micro simulation modeling study. Arch Iran Med. (2018) 21:213–22. 29738265

[25] GDP per Capita (current US$) - Iran Islamic rep. Data. Available online at: https://data.worldbank.org/indicator/NY.GDP.PCAP.CD?locations=IR (accessed January 21, 2023).

[26] Marseille E Larson B Kazi DS Kahn JG Rosen S. Thresholds for the cost–effectiveness of interventions: alternative approaches. Bull World Health Organ. (2014) 93:118–24. 10.2471/BLT.14.138206 25883405 PMC4339959

[27] Revill P Walker SM Madan J Ciaranello A Mwase T Gibb DM et al. Using Cost-effectiveness Thresholds to Determine Value for Money in Low-and Middle-income Country Healthcare Systems: Are Current International Norms fit For Purpose? York, UK: Centre for Health Economics, University of York (2014). (CHE Research Paper; 98).

[28] Woods B Revill P Sculpher M Claxton K. Country-Level cost-effectiveness thresholds: initial estimates and the need for further research. Value Health. (2016) 19:929–35. 10.1016/j.jval.2016.02.017 27987642 PMC5193154

[29] Esteghamati A Etemad K Koohpayehzadeh J Abbasi M Meysamie A Noshad S. Trends in the prevalence of diabetes and impaired fasting glucose in association with obesity in Iran: 2005–2011. Diabetes Res Clin Pract. (2014) 103:319–27. 10.1016/j.diabres.2013.12.034 24447808

[30] Noshad S Afarideh M Heidari B Mechanick JI Esteghamati A. Diabetes care in Iran: where we stand and where we are headed. Ann Glob Health. (2016) 81:839. 10.1016/j.aogh.2015.10.003 27108151

[31] Chamnan P Simmons RK Khaw KT Wareham NJ Griffin SJ. Estimating the population impact of screening strategies for identifying and treating people at high risk of cardiovascular disease: modelling study. BMJ. (2010) 340(apr23 2):c1693. 10.1136/bmj.c1693 20418545 PMC2859321

[32] Móczár C Rurik I. Comparison of cardiovascular risk screening methods and mortality data among Hungarian primary care population: preliminary results of the first government-financed managed care program. Slovenian J Public Health. (2015) 54:154–60. 10.1515/sjph-2015-0022 27646722 PMC4820151

[33] Schuetz CA Alperin P Guda S Herick AV Cariou B Eddy D. A standardized vascular disease health check in Europe: a cost-effectiveness analysis. PLoS ONE. (2013) 8:e0066454. 10.1371/journal.pone.0066454 23869204 PMC3712021

[34] Epstein D García-Mochón L Kaptoge S Thompson SG. Modeling the costs and long-term health benefits of screening the general population for risks of cardiovascular disease: a review of methods used in the literature. Eur J Health Econ. (2015) 17:1041–53. 10.1007/s10198-015-0753-2 26682549 PMC5047941

[35] Cutler RL. Fernandez-Llimos F, Frommer M, Benrimoj C, Garcia-Cardenas V. Economic impact of medication non-adherence by disease groups: a systematic review. BMJ Open. (2018) 8:e016982. 10.1136/bmjopen-2017-016982 29358417 PMC5780689

[36] Bansilal S Castellano JM Fuster V. Global burden of CVD: focus on secondary prevention of cardiovascular disease. Int J Cardiol. (2015) 1:S1–7. 10.1016/S0167-5273(15)31026-3 26747389

[37] Kim DD Wilkinson CL Pope EF Chambers JD Cohen JT Neumann PJ. The influence of time horizon on results of cost-effectiveness analyses. Expert Rev Pharmacoecon Outcomes Res. (2017) 17: 615–23. 10.1080/14737167.2017.1331432 28504026

