# Supplementary material for: A cost-effectiveness analysis of risk-based intervention for prevention of cardiovascular diseases in IraPEN program: A modeling study
Source: Front Public Health. 2023 Feb 24;11:1075277. doi: 10.3389/fpubh.2023.1075277 (PMC9999709; doi:10.3389/fpubh.2023.1075277)
Supplement: Supplementary file 1 [file Data_Sheet_1.pdf]

Supplementary Material

A COST-EFFECTIVENESS ANALYSIS OF RISK-BASED INTERVENTION FOR PREVENTION OF CARDIOVASCULAR DISEASES IN IraPEN PROGRAM: A MODELLING STUDY

Amirparviz Jamshidi[1, 2], Rajabali Daroudi[3], Eline Aas[2], Davood Khalili[1, 4]

1. Prevention of Metabolic Disorders Research Center, Research Institute for Endocrine Sciences, Shahid Beheshti University of Medical Sciences, Tehran, Iran
2. Department of Health Management and Health Economics, University of Oslo, Oslo, Norway
3. Department of Health Management, Policy and Economics, School of Public Health, Tehran University of Medical Sciences, Tehran, Iran
4. Department of Biostatistics and Epidemiology, Research Institute for Endocrine Sciences, Shahid Beheshti University of Medical Sciences, Tehran, Iran

* Corresponding Author

Davood Khalili, Prevention of Metabolic Disorders Research Center, Research Institute for Endocrine Sciences, Shahid Beheshti University of Medical Sciences, Tehran, Iran

Address: No. 23, Arabi st, Yaman st, Velenjak, Postal code: 1985717413

P O Box: 19395-4763, Phone: +98 21 22432500, Fax: +98 21 22416264

Cell no: +989128148865

Email: dkhalili@endocrine.ac.ir

TRANSITION PROBABILITIES

The model includes of 7 transition probabilities as below:

- _tp1: Annual stroke incidence rate
- _tp2: Annual CHD incidence rate
- _tp3: Stroke fatality rate for the first year
- _tp4: CHD fatality rate for the first year
- _tp5: non-CHD, non-stroke mortality rate
- _tp6: Fatality rate in stroke survivors
- _tp7: Fatality rate in CHD survivors

Except _tp3 and _tp4 that are constant through the model, the rest are time dependent transition probabilities. Therefore, as the model ages, these age-dependent transitions increase. With the starting point of 40 years old, _tp1 and _tp2 changes in every ten-year interval while _tp5, _tp6 and tp7 vary in every five-year interval.

Supplementary Figure 1 - The structure of the Markov model with transition probabilities

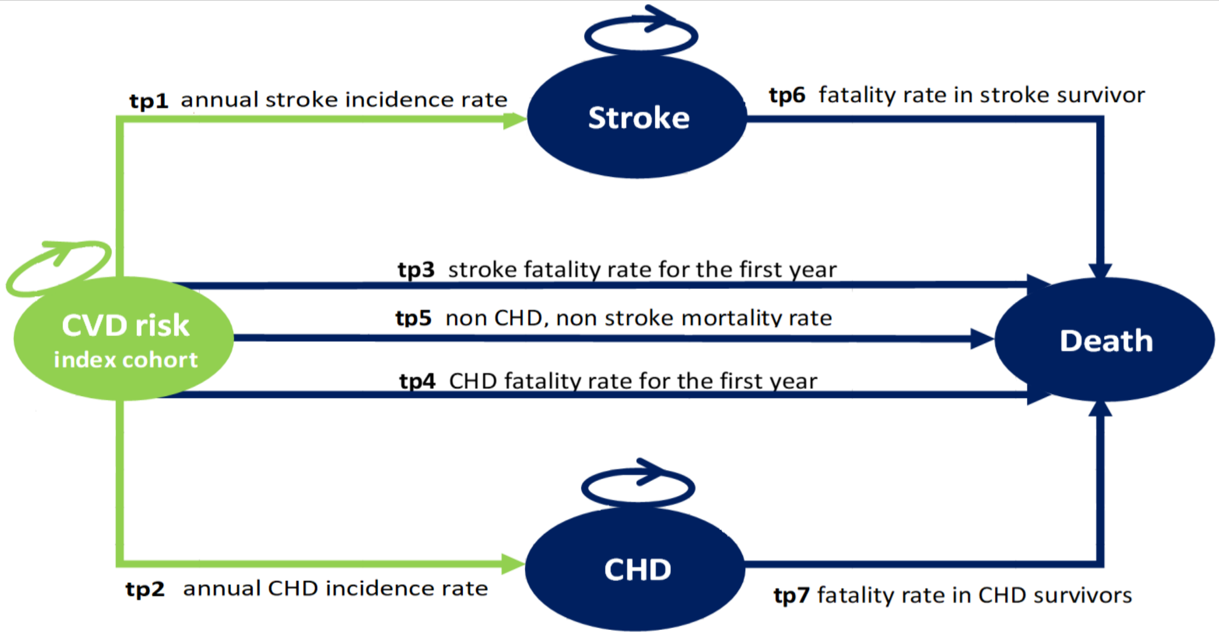

The annual incidence rate for coronary heart disease (CHD) and stroke were calculated from the Framingham study equations. Although the annual CVD-event rates table for Iranian population

was available, it could not be applied in this analysis as those rates were combined for all CVD events (Stroke and CHD together). Moreover, as 4 index cohorts had been defined with specific characteristics there was a need to calculate the risk based on those profiles. For calculating the annual risk of CHD, the risk factors below should be applied in the Framingham equation:

- Age
- Sex
- Systolic blood pressure (SBP)
- Total cholesterol and HDL
- Smoking and diabetes status
- Presence or absence of left ventricle hypertrophy (LVH)

As the HDL test and LVH detecting by electrocardiography (ECG) are relatively costly procedures, these two are not recommended by WHO PEN and are consequently not applied in CVD risk calculations of IRAPEN program. Therefore, it is assumed that none of the individuals has LVH. For HDL, according to available Iranian data, the averages of 50th percentile level of HDL were applied for all. Accordingly, for the women in all age groups 46 mg/dl was used and for the men 40 mg/dl except for men older than 55 that 41mg/dl was applied.[1] The CHD Framingham equation was formulated to calculate the CVD risk for people between 30 to 75 years old so it is not possible to calculate the rate for people older than 75 years old. The probabilities for men were gained as follows[2]:

$$a = 11.1122 - 0.9119 * \log(SBP) - 0.2767 * smoking - 0.7181 * \log(cholesterol/HDL) - 0.5865 * ECG\text{-}LVH$$

$$m_{male} = a - 1.492 * \log(age) - 0.1759 * diabetes$$

$$\mu = 4.4181 + m$$

$$\sigma = \exp(-0.3155 - 0.2784 * m)$$

[1] Hosseini, M, Navidi, I, Yousefifard, M. Serum HDL-C level of Iranian adults: results from sixth national Surveillance of Risk Factors of Non-Communicable Disease. J Diabetes Metab Disord. [Online] 2014;13(67): . Available from: https://www.ncbi.nlm.nih.gov/pmc/articles/PMC4099153/ [Accessed 9 May 2019].

[2] Andreson, K, Wilson, P, Odell, P, Kannel, W. An Updated Coronary Risk Profile A Statement for Health Professionals.Circulation. 1991;83(1): 356-362.

$u = (\log(t) - \mu) / \sigma$

$p = 1 - \exp -e^u)$

Therefore, for example, for a man 44 years old with SBP=130 mmHg, total cholesterol 195 mg/dl, HDL 40 mg/dl who does not smoke and is not diabetic, the 5-year probability is 0.024373. Although the risk is increased by age, it was assumed that this prediction is equally distributed through 5 years so it is divided by 5 to obtain the annual probability. These probabilities for women were calculated as follows:

a =11.1122-0.9119*log(SBP)-0.2767*smoking-0.7181*log(cholesterol/HDL)-0.5865*ECG-LVH

m_{female} = a - 5.8549 +1.8515* [log(age/74)]^2 -0.3758 * diabetes

$\mu = 4.4181 + m$

$\sigma = \exp(-0.3155 - 0.2784 * m)$

$u = (\log(t) - \mu) / \sigma$

$p = 1 - \exp -e^u)$

Supplementary table 1 – Annual incidence rate for CHD according to defined index cohorts

	**Low risk**	**Moderate risk**	**High risk**	**Very High risk**
Without diabetes				
**40-49**	0.00488	0.01139	0.01412	0.02836
**50-59**	0.00946	0.02006	0.02056	0.04231
**60-69**	0.01528	0.02160	0.02264	0.05586
**70-79**	0.02195	0.02297	0.02405	0.06837
With diabetes				
**40-49**	0.00526	0.01374	0.02808	0.03610
**50-59**	0.01037	0.02006	0.03633	0.05164
**60-69**	0.01369	0.02520	0.04909	0.06606
**70-79**	0.01470	0.02671	0.06118	0.07891

For calculating the annual risk of Stroke, the risk factors below should be applied in the Framingham equation[3]:

- Age
- Sex
- Systolic blood pressure (SBP)
- History of cardiovascular disease
- Smoking and diabetes status
- Presence or absence of left ventricle hypertrophy (LVH)
- Presence or absence of atrial fibrillation

As in IraPEN program LVH and atrial fibrillation are not detected, in addition, as the prevalence of these disorders in general population are less than 5%, it is assumed that none of the individuals suffer from these twos, consequently in the equation, both of them were put as 0.

L = 0.0505* Age + 0.0140*SBP + 0.3263*Hyp Rx+ 0.3384*DM+ 0.5147*Cigs + 0.5195*CVD + 0.6061*AF + 0 .8415*LVH

M = 0.0505*Ageµ + 0.0140*SBPµ + 0.3263*Hyp Rxµ+ 0.3384*DMµ+ 0.5147*Cigs µ+ 0.5195*CVDµ + 0.6061*AFµ +0 .8415*LVHµ

A = L - M

B = e^A

p = 1 - (S(1))^B Probability of stroke within 1 years

Hence for example, for a woman 54-year-old who smokes and does not have diabetes with systolic blood pressure of 150 mm/Hg, the annual transition probability for stroke event is 0.00134.

[3] Wolf, P, D'agostino, R, Belanger, A, Kannel, W. Probability of Stroke: A Risk Profile From the Framingham Study.Stroke by the American Heart Association. 1991;22(3): 312-318

Supplementary table 2 - Annual incidence rate for stroke according to defined index cohorts

	**Low risk**	**Moderate risk**	**High risk**	**Very High risk**
Without diabetes				
**40-49**	0.00102	0.00069	0.00103	0.00393
**50-59**	0.00168	0.00134	0.00198	0.00651
**60-69**	0.00279	0.00257	0.00381	0.01076
**70-79**	0.00461	0.00496	0.00735	0.01776
**>80**	0.00763	0.00954	0.01412	0.02926
With diabetes				
**40-49**	0.00047	0.00070	0.00417	0.00551
**50-59**	0.00091	0.00135	0.00690	0.00911
**60-69**	0.00176	0.00261	0.01140	0.01506
**70-79**	0.00340	0.00503	0.01882	0.02482
**>80**	0.00654	0.00969	0.03099	0.04080

The studies showed that these two equations have acceptable predictive ability for the Middle Eastern people.[4] For the rest of the transition probabilities, the Iranian data were employed in the model.

The data of different **medications' effects** and **states' utilities** were driven from western countries literature.

[4] Koohi F, Steyerberg EW, Cheraghi L, Abdshah A, Azizi F, Khalili D. Validation of the Framingham hypertension risk score in a middle eastern population: Tehran lipid and glucose study (TLGS). BMC Public Health. 2021 Apr 24;21(1):790. doi: 10.1186/s12889-021-10760-6. PMID: 33894756; PMCID: PMC8070324.
